# Medical Items

**Published:** 1896-07

**Authors:** 


### MEDICAL ITEMS.

GOLOWKOW announces in *Wratsch*, No. 7, that he has demonstrated beyond a doubt that the cholera vibrio can penetrate the shell and enter hens' eggs.

GEORGIA barbecues are no doubt very enjoyable by the Hoi polloi of the Association, but the reaction therefrom is unfavorable to business.—*St. Louis Clinique.*

THE working men of Birmingham, England, have endowed the hospitals of that city with a sum amounting to nearly two million dollars. In this good work they stand alone, no other city's working population having done so.

Mr. F. Haas, pharmacy Fifth avenue and Thirty-ninth street, and Fourth avenue and Twenty-first street, New York, has the distributing depot for S. H. Kennedy's Oak Extracts (Quercus alba). Mr. Haas is personally known to the business manager of this JOURNAL.

A LIVERPOOL physician makes a portable spirit lamp out of a thermometer case, by simply fitting it with a few strands of lamp cotton and then filling with spirits. Screw on the top and place a piece of rubber tubing over the joint, making it spirit tight. Good for sterilizing needles. A suitable companion to Pavy's urinary test case and for other purposes.

THE authorities in India have forbidden the use, by the natives, of the water bottle made of skin for transporting drinking-water, and have supplied metal buckets instead. They also require all water to be boiled before being drunk. These precautions are instituted to prevent a return of the virulent epidemic of cholera that prevailed last year.—*Med. News.*

---

CHANGES IN THE ATLANTA COLLEGES.—Quite a large number of changes have been made in the Faculties of the two Medical Colleges here. As it is difficult to give them in detail, readers are referred to advertising pages xxii. and xxiii. for information as to vacancies filled, positions changed and additions made in the faculty lists and corps of assistants.

---

M. ROULIN, relying upon clinical experience and struck by the dangers of Roux's serum, proposes to reserve its use for those cases in which the lesions are not accessible. Otherwise he prefers to treat patients by local douches of water charged with carbolate of soda which he has long used with much success. (Translated from *La Médecine Moderne,* May 23, 1896.)

---

CAPPELNI (*Norsk Magazine for Lægevidenskaben and British Medical Journal,* May 23, 1896) reports a man struck by a knife which wounded a large branch of the coronary arteries and made a cut two inches long in the left ventricle after passing through the pleura without touching the lung. The ventricle was stitched, and the man lived for two and a half days. The necropsy showed pericarditis and various bacteria in the fibrinous exudation.

---

We have been requested to announce that Professor Dr. Don Francisco Bastillos, Calle de Tacuba, No. 7, Ciudad de Mexico D. F. Republica Mexicana, has been elected Treasurer of the Second Pan-American Medical Congress, to be held in the city of Mexico, beginning the 16th of November. All members residing in the United States and Canada, and others, who contemplate attending, should forward the registration fee, $5.00, gold, to him at once, and notify Dr. C. A. L. Reed, Cincinnati.

Do not use the old-fashioned curved bistoury in opening the simplest abscess. It is unsurgical because you proceed from within outward—from the unknown to the known. This is a false principle in philosophy, in surgery, in everything. Cut from the surface inward and you can deal with difficulties in the order in which they occur. Always work with the aid of sight, and do not pin your faith on anatomy.—*Internat. Jour. Surg.*

---

"I DARE any political economist to show me one expedient whereby conception may be avoided. I challenge him to name a single preventive which will not do damage either to good health or good morals. Even natural sterility is a curse. Show me a home without children, and ten to one you show me an abode dreary in its loneliness, disturbed by jealousy and estrangement, distasteful from wayward caprice or from unlovable eccentricity." —*Dr. Wm. Goodell.*

---

THE death, at the age of sixty-eight, of Sir J. Russell Reynolds, M.D., F.R.C.P., took place in London on May 29th. He was one of the best known of English physicians, and had held many positions of honor and importance. He was best known in this country as the editor of his "System of Medicine," but his chief work was as a neurologist. He wrote among other things "Diagnosis of Diseases of the Brain, Spinal Cord and Nerves," "Tables for Diagnosis of Diseases of the Brain." An essay on "Vertigo," and "Scientific Value of Legal Tests of Insanity."

---

DR. J. G. CLARK reports (*Maryland Medical Journal*, May 30, 1896) that for the last two years very beneficial effects in diminishing the thirst, so common and so trying to patients after abdominal operations, have been obtained by injecting one litre of normal salt solution into the rectum before the cessation of anesthesia. The patient is elevated to the medium high Trendelenburg posture, a stiff rectal tube is inserted well up into the sigmoid flexure and the fluid slowly poured into the glass funnel, which is held three or four feet above the level of the patient's buttocks.

---

THE death of Germain Sée, already announced, removes a well-known figure from Parisian medical life and an earnest and successful worker from among the older body of investigators. He was born at Ribeauville, on the Rhine, in 1818, of a Jewish family. Besides his numerous contributions on the subjects of the blood, nerves, asthma, the heart, rheumatism, the lungs, stomach, etc., he is well known in Paris as the man who did most there for the introduction to general use of such drugs as salicylate of soda, antipyrin, sparteine, creosote, iodide of potassium, etc., etc.

---

THE following contains the gist of a paper in the June volume of the *New Orleans Medical and Surgical Journal* by Dr. Just Touatre: "*If it be true* that the sulphate of quinine is all-powerful in acute paludism, that mercury and iodide of potassium succeed in checking the secondary and the tertiary accidents of syphilis, that the salicylate of sodium is the specific remedy for acute rheumatism, and colchicum seed that for acute gout, etc., *then it is also true* that oxygen is the most efficacious remedy in diseases of the blood. . . . *These inhalations of oxygen increase and revivify the red corpuscles, multiply the leucocytes, oxidize the toxins, augment their solubility*, and, therefore, *facilitate their elimination.*"

---

NONE of the female sexual organs are usually thought to suffer from medical neglect, but Dr. Buford (*Memphis Medical Monthly*, June) read a paper recently before the Memphis Medical Society in which he reminded his audience that the clitoris is a frequent focus of disease. Through irritation due to adhesion between the clitoris and the surrounding mucous membrane various reflex ailments are set up, as well as the tendency to local disturbances which often result in evil habits. Buford directs attention to the advisability of strict cleanliness and of the more frequent examination of this organ.

Lack of sexual feeling in the female has been cured by another physician in several instances by freeing the clitoris from pathlogical adhesions.

---

*Le Progrès Médical* (May 13, 1896), contains a report of a paper read on May 4th by Dr. Lucien Howe, of Buffalo, N. Y., before the French Ophthalmological Society upon the laws of the United States concerning the treatment of ophthalmia neonatorum. Dr. Howe was last year elected President of the Ophthalmological Section of the American Medical Association, but was unable to be present in Atlanta. Drs. Terson, Garecki, Abadie, Galezowski, Cleibret, and Parinaud took part in the discussion which followed the reading of the paper. Opinion was divided concerning the results of such laws in France, but the speakers generally advised the training of nurses and midwives in order that they might recognize the seriousness of these cases and treat them till they could be carried to the specialist.

---

WE are requested by Dr. Sternberg, Surgeon-General U. S. Army, to announce that there are at present three vacancies in the Medical Corps of the U. S. Army, and it is expected that at least three more will occur during the present year. As usual an Army Medical Board will meet in Washington early in October for the examination of candidates. Permission to appear before the board is obtained by letter to the Secretary of War, which must be in the handwriting of the applicant, giving the date and place of his birth and the place and State of which he is a permanent resident, and inclosing certificates, based on personal acquaintance, from at least two reputable persons as to his citizenship, character, and habits. For further requirements of admission, address the Secretary of War, Washington.

